# Watershed‐scale controls outweigh local crossing effects on sediment loss from unpaved roads

**DOI:** 10.1002/jeq2.70138

**Published:** 2026-01-21

**Authors:** Kathleen J. Cutting, Shannon L. Speir, Alana G. Strauss, Karessa G. De La Paz, Caroline G. T. Anscombe

**Affiliations:** ^1^ Department of Crop, Soil, and Environmental Sciences University of Arkansas Fayetteville Arkansas USA; ^2^ University of Arkansas Division of Agriculture Little Rock Arkansas USA

## Abstract

In rural areas, unpaved roads can drive water quality degradation via sediment inputs. Excess sediment loss from poorly maintained unpaved roads to adjacent waterways blocks sunlight, decreasing primary productivity and increasing nutrient concentrations. This is particularly relevant to Arkansas, where 85% of county roads are unpaved; however, few studies have explored the impacts of unpaved roads in rural watersheds dominated by pasture. We sampled Brush Creek (Arkansas) to understand local (i.e., road crossing type) and watershed‐scale (e.g., land cover/use) controls on sediment loss. We collected monthly baseflow and four opportunistic storm flow samples for total suspended solids (TSS) upstream and downstream at bridge, culvert, and direct stream crossings. Mean TSS yields downstream versus upstream of road crossings were comparable, especially at bridge and culvert sites, indicating these road crossings may not be critical TSS sources. At the watershed scale, TSS load showed increasing trends as both total length of unpaved roads and area of pastureland in a subwatershed increased (linear mixed effects; *β* = 0.03, *R* ^2 ^= 0.41, *p* > 0.1; *β* = 0.67, *R* ^2 ^= 0.42, *p* = 0.07, respectively). Moreover, TSS yields were higher during stormflow than baseflow (26.87 ± 6.82 vs. 0.38 ± 0.04 kg km^−2^ day^−1^; unpaired t‐test, *p* < 0.01). Finally, seasonality influenced local and watershed patterns of TSS loss via variation in transport controls, including wet season conditions, discharge rates, and overland flow. Our findings indicate watershed‐scale characteristics are key contributors to sediment loss in rural watersheds. Targeted best management practice implementation should focus on unpaved roads and pasturelands during key transport periods to effectively protect downstream water quality.

## Core Ideas

- Periods of stormflow are key times of sediment loss.
- Road crossing type and up‐ versus downstream of crossings minimally impacted sediment yields.
- Watershed‐scale factors, like unpaved roads and pastureland, typically increase sediment loads.

**Abbreviations**
ANOVA: analysis of variance
BMP(s): best management practice(s)
DS: downstream
LME: linear mixed effects
TSS: total suspended solids
US: upstream

## 1 INTRODUCTION

Sediment is a leading pollutant to our freshwater systems as a result of rapid land use and land cover change (Worku et al., 2017). Runoff containing sediments, measured as total suspended solids (TSS), is largely dependent on precipitation events that increase erosion and connectivity across the aquatic–terrestrial interface (Allan et al., 2021; Krishnappan et al., 2009; Rose & Karwan et al., 2021). Excess sediment loss to adjacent waterways can block sunlight to the water column, decreasing primary productivity and increasing contaminant and nutrient concentrations, negatively impacting freshwater ecosystem function and services (Wood & Armitage et al., 1997). Although concentrations of sediment in homogeneous agricultural and urban areas have decreased over time as a result of land management changes (Murphy, 2020), sediment erosion from unpaved roads and road crossings is often overlooked as a continued key source of sediments to streams (Farias et al., 2021; Lane & Sheridan, 2002).

Unpaved roads, which are prevalent in rural areas across the United States, are one of the largest anthropogenic sources of fine sediments to streams (Silliman & Toman, 2019) due to traffic traveling over unpaved roads grinding down sediments (Alvis et al., 2023), poorly maintained roads (Clinton & Vose, 2003), and roadside ditches (Wemple et al., 2017). In fact, fine sediments typically make up the majority of suspended sediments often transported from nonpoint source pollution, such as unpaved roads (Church, 2006; Krishnappan et al., 2009; Wilcock et al., 2009). These fine sediments from unpaved roads are easily eroded, especially during precipitation events as overland flow travels over these roads (MacDonald et al., 2001; Ramos‐Scharrón & LeFevor, 2016; Ziegler et al., 2004).

Streams are particularly vulnerable to sediment erosion from unpaved roads where roads cross the stream. As such, best management practices (BMPs) on unpaved roads and their crossings, like the use of bridges and properly sized culverts, broad‐based dips, and grassed ditches, can reduce sediment loads (Keller & Sherar, 2003). Bridges and culverts prevent streambed disturbance from vehicles while allowing streams to follow a natural flow path (Keller & Sherar, 2003; Morris et al., 2016). While fords or direct water crossings may serve as a more cost‐effective type of road crossing (Morris et al., 2016), they can increase erosion, streambed disturbances via traffic, and increase maintenance costs due to frequent washouts and rutting (Morris et al., 2016; Sample et al., 1998). Moreover, proper implementation of stream crossing BMPs and regular road maintenance is essential to reduce the transport of excess sediment loads to nearby streams (Wemple et al., 2017). This is critical as unpredictable, large precipitation events are increasing under climate change (Lu et al., 2020; Thackeray et al., 2022; Wasko et al., 2021), putting unpaved road BMPs at risk of failure increasing downstream (DS) sediment loss (Bhatkoti et al., 2016; Jaeger, 2020; Wilhere et al., 2017). Given the widespread prevalence of unpaved roads in rural areas, it is essential to monitor unpaved road networks to understand their impact on water quality.

**Core Ideas**

- Periods of stormflow are key times of sediment loss.
- Road crossing type and up‐ versus downstream of crossings minimally impacted sediment yields.
- Watershed‐scale factors, like unpaved roads and pastureland, typically increase sediment loads.

In Arkansas 85% of county roads are unpaved, and unpaved roads are the second largest nonpoint source of pollution in the state (Arkansas Department of Agriculture Division of Natural Resources, 2024). The Northwest Arkansas region is dominated by pasturelands and poultry farming (USDA, 2018), where many areas are highly trafficked by large vehicles, amended with poultry litter, and potentially disturbed by overgrazing and soil compaction from livestock (Katuwal et al., 2023; Mulholland & Fullen, 1991; Pilon et al., 2017; Roman et al., 2021). All of these factors may interact to contribute to increased sediment loss from the landscape. Since 2015, the Arkansas Unpaved Roads Program has worked to reduce sediment loss through education and implementation of BMPs on public lands (Arkansas Department of Agriculture Natural Resources Division, 2023). However, we still lack an understanding of how unpaved road networks and varying road crossings impact sediment transport in more heterogeneous landscapes (Murphy, 2020; Wemple et al., 2017), as most previous research on unpaved roads has primarily been conducted in forestry settings (Alvis et al., 2023; Lane & Sheridan, 2002; Kastridis, 2020); few studies have explored the impacts of unpaved roads on rural watersheds dominated by pasture.

Here, the objective of our study was to understand local and watershed‐scale controls on sediment loss from unpaved roads in the Brush Creek watershed, Arkansas. We conducted synoptic sampling of Brush Creek, Arkansas, during baseflow and stormflow from February 2024 to January 2025 to address the following research questions:

1. Do locations DS of road crossings demonstrate higher sediment yields than locations upstream (US) thereof?
  - We hypothesized that DS versus US of a road crossing will increase TSS yields as sediment is disturbed by vehicles traveling over roads entraining sediment DS (Boggs et al., 2018).
2. Does the type of road crossing (e.g., bridge, culvert, and direct stream crossing) impact sediment loss from unpaved roads?
  - We hypothesized that direct stream crossings will have higher TSS yields compared to bridges and culverts as the sediment travels a shorter distance from the road to enter the stream (Clinton & Vose, 2003).
3. Does the extent of unpaved roads and the land use within subwatersheds impact sediment loss in Brush Creek?
  - We hypothesized that higher lengths of unpaved roads in subwatersheds will increase sediment loads as overland flow travels over unpaved road networks (Ramos‐Sharrón & LeFevor, 2016).
  - We hypothesized that subwatersheds with greater area of pasturelands will have higher TSS loads as poultry litter amended fields and disruption of soil from livestock create sources of easily transportable sediment with overland flow (Mulholland & Fullen, 1991; Pilon et al., 2017).

Our results will help inform land managers on where to prioritize BMPs to reduce sediment loss in rural heterogeneous areas with a high presence of unpaved roads under a changing climate.

## 2 METHODS

### 2.1 Study site

**FIGURE 1:**
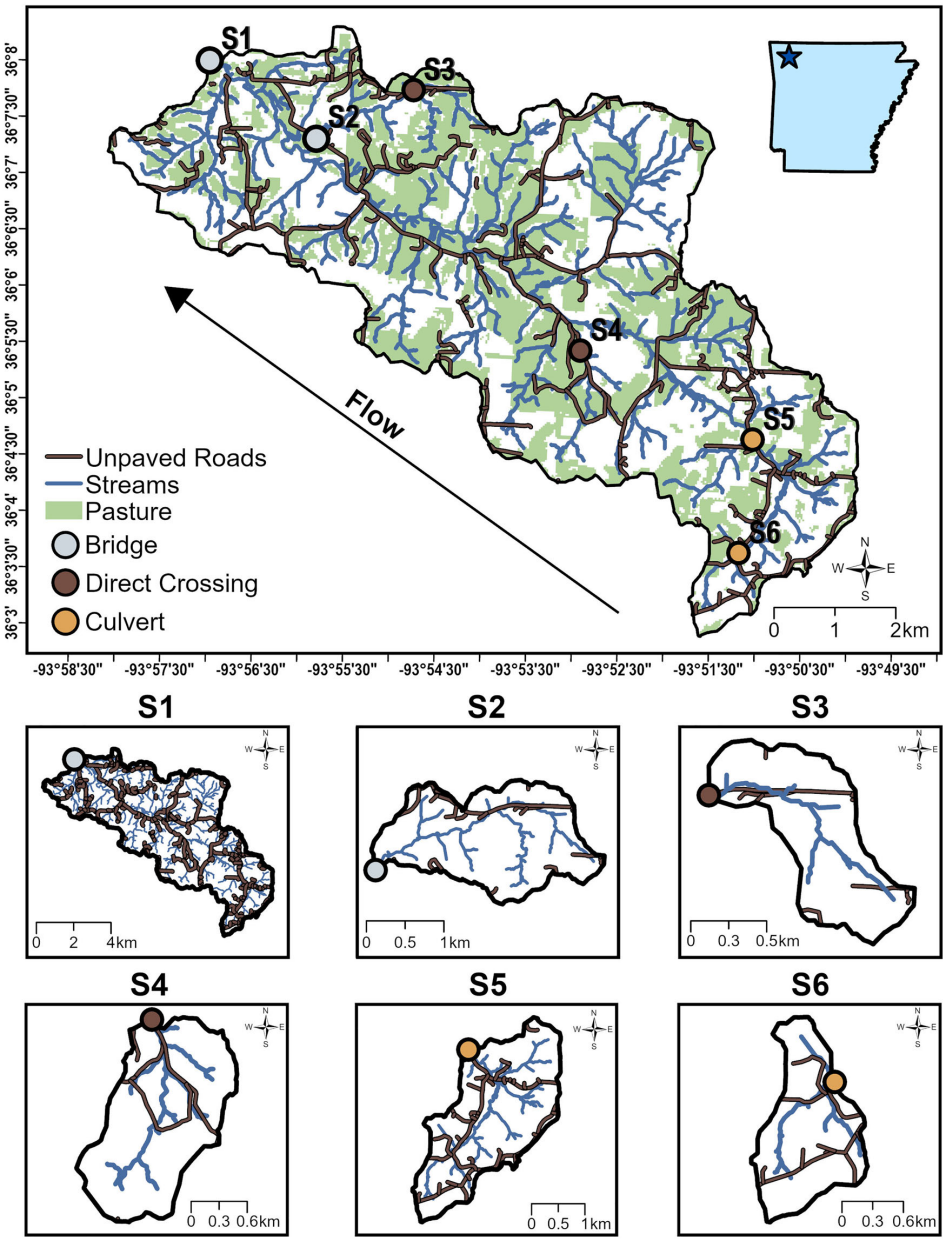
The Brush Creek watershed, with site S1 as the outlet and increasing in number moving upstream. Each site was sampled upstream and downstream of the road crossing. Bridge crossings are shown in gray, direct crossings in brown, and culvert crossings in orange. Brush Creek has 22 km^2^ of pasture or hay area. The subwatershed for each site is shown in the bottom half of the figure.

Brush Creek is a non‐perennial, partial HUC12 tributary of Beaver Lake Reservoir, the primary drinking source in northwest Arkansas (Figure 1; Ouei & Daniels, 2017). Brush Creek is a third‐order stream, with a mean flow of 517.7 L s^−1^ at the watershed outlet. The wet season in Brush Creek normally occurs in spring and winter; however, it is important to note there was an intense flow event at the end of the dry season (November; Table 1). Brush Creek is 5165 ha and hosts a variety of land use and management. Brush Creek has approximately 191 km of unpaved roads and 22 km^2^ of pasture area (Table 2; Figure 1). The predominant forms of agriculture in the watershed are beef cattle and poultry production (*Gallus gallus domesticus* and *Meleagris gallopavo*). In Brush Creek, there are 76 poultry houses and 33 of 745 parcels have beef cattle present. Beef cattle are excluded from entering streams at the majority of sampling sites, but we have observed beef cattle in the stream at two sites (S2 and S3). We were unable to obtain detailed information regarding the maintenance of unpaved roads in Brush Creek; however, there is a “county maintenance ends” sign near the watershed outlet. Additionally, we have observed road maintenance through gravel application and road flattening and have discovered that road maintenance is privately conducted based on discussions with local landowners. In Brush Creek, culverts are made from concrete or metal, and bridges are made of concrete with gravel and sediment across the top.

**TABLE 1:** Total precipitation in each sampling month and from each storm event. We defined any precipitation 48 h prior to the storm event and the precipitation the day of the storm event as total precipitation for each storm. We collected precipitation data from National Oceanic and Atmospheric Administration (NOAA) weather data at the Fayetteville Drake Field station.

Month	Total precipitation
MM/YYYY	cm
2/2024	3.0
3/2024	9.5
4/2024	10.9
5/2024	12.3
6/2024	4.8
7/2024	15.2
8/2024	15.2
9/2024	0.4
10/2024	3.5
11/2024	23.8
12/2024	7.3
1/2025	4.5
Storm date	
March 15, 2024	1.4
May 6, 2024	3.0
July 9, 2024	2.2
November 5, 2024	18.6

**TABLE 2:** Land use at each site in the Brush Creek watershed. Sites are listed from downstream to upstream and are defined by their road type crossing. Starred sites indicate that upstream and downstream land use characteristics differed based on differing watersheds; values here are the average of upstream and downstream.

			Land use	
Site	Road crossing type	Watershed area (km^2^)	Length of unpaved roads (km)	Pasture/hay area (km^2^)
S1	Bridge	51.6	190.8	22.5
S2	Bridge	3.0	8.2	1.7
S3*	Direct	1.2	3.5	0.9
S4	Direct	2.3	7.4	1.1
S5	Culvert	5.6	31.2	1.8
S6*	Culvert	1.3	8.5	0.4

In addition, Brush Creek is located in the Boston Mountains and Ozark Highlands ecoregions (Figure S1; US Environmental Protection Agency, 2015). The soil in Brush Creek is made up of majority Enders–Leesburg complex, 8%–20% slopes (20.1%), Enders–Leesburg complex, 20%–40% slopes (7.2%), Noark very gravelly silt loam, 20%–40% slopes (7.2%), and Nixa very gravelly silt loam, 3%–8% slopes (6.3%) (Table S1). In addition, the primary soil hydrologic group type is D—soils having very slow infiltration rates consisting of soils that are either clay, have a high water table, or are shallowly located above impervious material (USDA, 2017). The Boston Mountains are underlain by Pennsylvanian sandstone, shale, and siltstone. The Ozark Highlands, specifically the Springfield Plateau where part of Brush Creek is located, is dominated by karst features such as caves, sinkholes, and underground drainage, impacting water quality as there is high permeability to groundwater (Legrand & Stringfield, 1973; Woods et al., 2004). In addition, spring‐fed streams are common in the Springfield Plateau. Overall, water quality in the Boston Mountains reflects the geology, soils, and land use, whereas water quality in the Ozark Highlands is strongly influenced by lithology and land use practices (Woods et al., 2004).

### 2.2 Field methods

We took monthly grab samples at baseflow at three mainstem sites and three tributary sites in Brush Creek from February 2024 to January 2025 (Figure 1). Here, we define baseflow sampling as at least 48 h following a storm or high‐flow event (Thompson et al., 2021). We categorized the sites into three types of road crossings: bridge, culvert, and direct stream crossings (*n* = 2 per crossing type). We selected sampling sites based on accessibility and availability of road type crossing but recognize that site S1 is the watershed outlet and introduces a scale limitation in the study. We then collected samples at flowing sites, starting with the most DS site (site S1) and moving US through the watershed. At each site, we sampled using a paired sampling approach, sampling DS and then US of the road crossing. We sampled a minimum of 10 m DS and US of the road crossing; some sites were sampled 15 m from the road crossing if site characteristics allowed. Sample location depended on the characteristics of each site to ensure a well‐mixed sample (e.g., tributary inlets, accessibility, etc.). At sites deep enough to prevent streambed disturbance, we collected water using a 4‐L sample bottle by triple rinsing the bottle with site water before collecting an unfiltered sample. To avoid disturbance of the benthos at shallow sites, we used a smaller sample bottle (also triple rinsed) to collect site water that was used to triple rinse and fill the 4‐L sample bottle. Additionally, we sampled four storm events to capture TSS loss under a range of flow conditions in Brush Creek. We sampled storm events within 24 h of the cessation of peak precipitation (Table 1). We collected precipitation data from National Oceanic and Atmospheric Administration (NOAA) weather data at the Fayetteville Drake Field station. All samples were stored on ice for transport to the laboratory, where they were stored in the refrigerator until analysis.

At each sampling location, we also measured stream discharge using the cross‐sectional area method using a flow meter (OTT HydroMet) and stadia rod at either US or DS of the road crossing or both if there was an additional source of water entering the stream, such as a road ditch (Hauer & Lamberti, 2011).

### 2.3 Laboratory methods

We analyzed the stored water samples within 24 h of collection using gravimetric analysis (Hauer & Lamberti, 2017). To prepare our filters, we pre‐filtered 100 mL of deionized water through a 0.22‐µm glass microfiber filter (GF/F; Millipore). The filter was then dried at 50°C for at least 48 h and weighed to collect a filter weight (in g). We then filtered our field samples through the prepared and weighed filters until the filters clogged, measuring the exact volume filtered. After filtering, we dried the filters in a drying oven at 50°C for 72 h. After 72 h, the filters were cooled, and we reweighed each filter to measure the TSS residue to determine the TSS concentration.

### 2.4 Calculations

We calculated the overall TSS concentration (*C*, in g L^−1^) at each site:

(1) $$C=\left(\mathrm{AFW}-\mathrm{PFW}\right)/\mathrm{TV}$$

where AFW is the post‐filtration filter weight (g), PFW is the pre‐filtration filter weight (g), and TV is the total volume of the water used in filtering (L). We then converted *C* to mg L^−1^ to utilize in our results.

We also calculated instantaneous TSS loads (as *C* × discharge [*Q*]; kg day^−1^), the mass of TSS passing over a point in the stream over a specific time interval, at each sampling site. We also normalized TSS loads by subwatershed area to calculate TSS yields (kg km^−2^ day^−1^).

Finally, we calculated change in TSS yields (ΔTSS, in kg km^−2^ day^−1^) between US and DS by subtracting US yield from DS yield:

(2) ΔTSS=Downstreamyield−upstreamyield

Here, positive values indicate greater DS yield, negative values indicate greater US yield, and zero values indicate no difference between TSS yields US and DS.

### 2.5 Geographic Information System methods

In ArcGIS Pro (3.2, Esri), we delineated subwatershed areas of each site using the hydrology toolbox of the spatial analysis module utilizing the NAD 1983 (2011) State Plane Arkansas (Meters) North coordinate system and United States Geological Survey (USGS) 10 m digital elevation model data, treating the sampling location as the pourpoint. Next, we used Multi‐Resolution Land Characteristics Consortium NLCD 2021 Conterminous United States land cover projected in the aforementioned coordinate system to find the land use area in each subwatershed. To find the total length of roads in each subwatershed, we used the Topologically Integrated Geographic Encoding and Referencing 2021 secondary roads shapefile for Madison and Washington Counties, Arkansas, mapped in ArcGIS Pro. To validate unpaved roads identified in the Geographic Information System, we confirmed the presence of unpaved road by conducting two windshield surveys during the study year. We then edited the unpaved roads shapefile layer to match the presence of unpaved roads in the Brush Creek watershed. Lastly, we clipped the unpaved roads layer to each site subwatershed where we found the total length of unpaved roads (km) in each subwatershed.

### 2.6 Statistical methods

We performed all statistical analyses using R Statistical Software (v4.3.1; R Core Team, 2023). To address *Hypothesis 1*, we used paired *t*‐tests to compare US and DS TSS yields across all sites separated for baseflow and stormflow. To address *Hypothesis 2*, we used a one‐way analysis of variance (ANOVA) to compare ΔTSS across bridge, culvert, and direct stream crossings. Finally, to address *Hypothesis 3*, we used linear mixed effects (LME) models to assess the relationship between TSS loads and pastureland area and total length of unpaved roads while accounting for storm events. Site was included as a random effect to account for repeated measures at the sampling locations. We removed site S1 from *Hypothesis 3* analyses to address the disparity in subwatershed size. All assumptions (i.e., normality, heteroskedasticity, independence, compound symmetry, sphericity, linearity, and outliers) were checked for each test, and a *p* < 0.05 was used as our alpha level. If data did not meet assumptions, we log‐transformed the data before conducting statistical tests.

## 3 RESULTS

### 3.1 Patterns in discharge drive sediment yield during baseflow and stormflow

**FIGURE 2:**
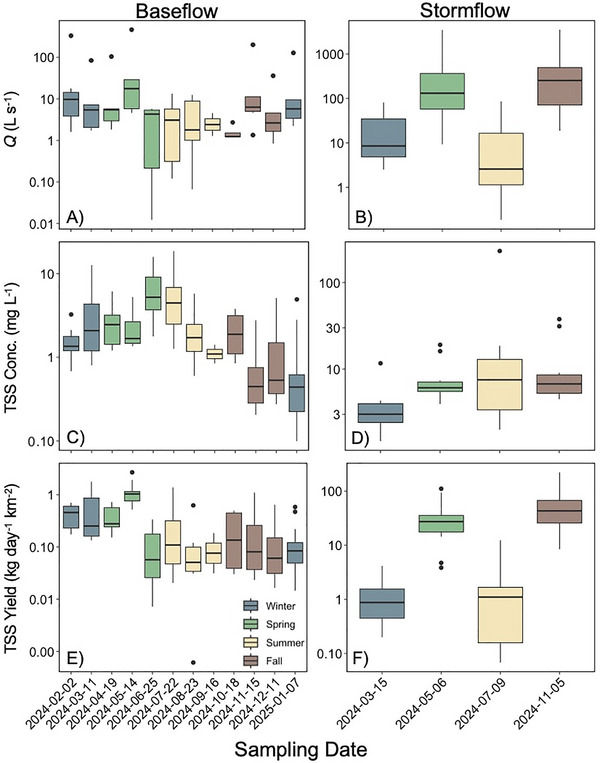
Discharge (*Q*), total suspended solids (TSS) concentration (mg L^−1^), and TSS yield (kg km^−2^ day^−1^) across the study period during baseflow (A, C, and E) and stormflow (B, D, and F). Note the *y*‐axis range varies based on flow condition and is shown on a log scale. Dates are listed in chronological order of sampling and are colored by season. For each date box plot, the central thick horizontal line indicates the median of the distribution, the box limits represent the upper (Q3) and lower (Q1) quartiles, the whiskers extend to 1.5 times the interquartile range (IQR) from the box, and points beyond the whiskers denote outliers represented by black circles.

At baseflow, mean discharge (*Q*) was greatest in May (mean ± standard error; 89.4 ± 50.3 L s^−1^) and lowest in October (1.6 ± 0.4 L s^−1^; Figure 2A). We documented a decrease in mean *Q* during the middle of the sampling period, corresponding with the dry period of the year (Figure 2A). Bridge sites had the greatest mean *Q* in the spring (100.0 ± 50.0 L s^−1^), whereas culvert sites had the lowest mean *Q* in the summer (0.7 ± 0.6 L s^−1^; Table 3). Overall, mean *Q* was significantly greater in winter (35.1 ± 13.3 L s^−1^) and spring (44.8 ± 21.2 L s^−1^) seasons compared to summer (4.3 ± 1.1 L s^−1^; ANOVA, *p* < 0.01; Table 3 and Table S2).

**TABLE 3:** Road crossing seasonal discharge (*Q*), TSS concentration, and yield (mean ± standard error [SE]).

		Baseflow			Stormflow		
Season	Road crossing	*Q* (L s^−1^)	TSS conc. (mg L^−1^)	TSS yield (kg km^−2^ day^−1^)	*Q* (L s^−1^)	TSS conc. (mg L^−1^)	TSS yield (kg km^−2^ day^−1^)
Winter	Bridge	94.1 ± 34.8	1.7 ± 1.0	0.4 ± 0.1	44.4 ± 21.1	2.2 ± 0.3	0.4 ± 0.1
	Culvert	6.5 ± 1.5	1.6 ± 0.5	0.2 ± 0.1	19.6 ± 8.5	3.7 ± 0.2	1.6 ± 0.2
	Direct	4.8 ± 1.0	2.6 ± 0.5	0.5 ± 0.1	6.2 ± 1.9	5.1 ± 2.3	1.6 ± 0.9
	Average	35.1 ± 13.3	2.0 ± 0.4	0.4 ± 0.1	23.4 ± 8.4	3.7 ± 0.8	1.2 ± 0.3
Spring	Bridge	100.0 ± 50.0	2.9 ± 0.7	0.7 ± 0.2	2341.8 ± 1134.2	14.0 ± 3.7	73.1 ± 29.8
	Culvert	10.5 ± 4.1	3.5 ± 0.7	0.5 ± 0.1	210.8 ± 88.2	6.0 ± 0.2	29.0 ± 3.8
	Direct	5.8 ± 1.9	4.5 ± 1.4	0.7 ± 0.2	83.8 ± 43.6	5.5 ± 0.7	17.5 ± 7.7
	Average	44.8 ± 21.2	3.3 ± 0.6	0.6 ± 0.1	745.8 ± 408.7	8.0 ± 1.5	36.8 ± 10.4
Summer	Bridge	7.1 ± 1.7	2.4 ± 0.5	0.1 ± 0.0	51.5 ± 20.2	7.4 ± 2.3	1.7 ± 0.0
	Culvert	0.7 ± 0.6	8.3 ± 3.1	0.1 ± 0.1	0.7 ± 0.4	11.9 ± 3.5	0.1 ± 0.0
	Direct	2.4 ± 0.8	4.3 ± 1.5	0.6 ± 0.3	2.6 ± 0.7	60.4 ± 56.9	3.7 ± 2.9
	Average	4.3 ± 1.1	4.3 ± 1.0	0.2 ± 0.1	18.3 ± 9.4	26.6 ± 18.7	1.8 ± 1.0
Fall	Bridge	49.7 ± 25.5	0.6 ± 0.1	0.1 ± 0.0	1869.1 ± 955.7	19.9 ± 8.6	117.8 ± 50.2
	Culvert	2.4 ± 0.9	1.2 ± 0.5	0.1 + 0.0	286.1 ± 126.6	6.3 ± 0.8	41.1 ± 10.5
	Direct	4.8 ± 1.2	1.7 ± 0.6	0.4 ± 0.1	192.0 ± 102.2	7.2 ± 0.9	46.4 ± 20.3
	Average	21.5 ± 10.5	1.2 ± 0.3	0.2 ± 0.1	748.4 ± 373.2	11.1 ± 3.2	68.4 ± 19.7

Mean baseflow TSS concentration was greatest in June and July (6.6 ± 1.5 mg L^−1^ and 5.9 ± 1.5 mg L^−1^, respectively) at baseflow and decreased in late fall, with the lowest mean TSS concentration measured in November (0.7 ± 0.2 mg L^−1^; Figure 2C). Culvert sites had the greatest mean TSS concentration in the summer (8.3 ± 3.1 mg L^−1^), and bridge sites had the lowest mean TSS concentration in the fall (0.6 ± 0.1 mg L^−1^; Table 3). Mean TSS concentration was significantly greater in spring (3.3 ± 0.6 mg L^−1^) and summer (4.3 ± 1.0 mg L^−1^) compared to winter (2.0 ± 0.4 mg L^−1^) and fall (1.2 ± 0.3 mg L^−1^; ANOVA, *p* < 0.001; Table 3 and Table S3).

At baseflow, mean TSS yield was greater during the wet season, and we measured the highest mean yields in May (1.1 ± 0.2 kg km^−2^ day^−1^; Figure 2E). Mean TSS yields decreased in June and remained relatively constant across the remainder of the sampling period (Figure 2E). We measured the lowest mean TSS yield in September (0.1 ± 0.1 kg km^−2^ day^−1^; Figure 2E). Bridge and direct stream crossing sites had the greatest mean TSS yield in the spring (0.7 ± 0.2 kg km^−2^ day^−1^), and bridge sites had the lowest mean TSS yield in the summer and fall (0.1 ± 0.0 kg km^−2^ day^−1^) with culvert sites having the lowest mean TSS yield in the fall (0.1 ± 0.0 kg km^−2^ day^−1^; Table 3). Seasonal patterns in mean TSS yield matched seasonal patterns in *Q*, where winter (0.4 ± 0.1 kg km^−2^ day^−1^) and spring (0.6 ± 0.1 kg km^−2^ day^−1^) were greater than summer (0.2 ± 0.1 kg km^−2^ day^−1^) and fall (0.2 ± 0.1 kg km^−2^ day^−1^; ANOVA, *p* < 0.01; Table 3 and Tables S2 and S4; Figure 2A,E).

In general, we measured higher mean Q, as well as TSS concentrations and yields during stormflow (Figure 2). We documented storms of similar magnitude in May (spring), November (fall), March (winter), July (summer; Table 3; Figure 2B). Therefore, mean Q was significantly greater in spring (745.8 ± 408.7 L s^−1^) and fall (748.4 ± 373.2 L s^−1^) than winter (23.4 ± 8.4 L s^−1^) and summer (18.3 ± 9.4 L s^−1^; ANOVA, *p* < 0.00; Table 3 and Table S2). Bridge sites had the greatest mean *Q* in the spring (2341.8 ± 1134.2 L s^−1^) and culvert sites had the lowest mean *Q* in the summer (0.7 ± 0.4 L s^−1^; Table 3). Mean TSS concentration was significantly greater in summer (26.6 ± 18.7 mg L^−1^) and fall (11.1 ± 3.2 mg L^−1^) seasons compared to winter (3.7 ± 0.8 mg L^−1^; ANOVA, *p* < 0.05; Table 3 and Table S3). Direct stream crossing sites had the greatest mean TSS concentration in the summer (60.4 ± 56.9 mg L^−1^), and bridge sites had the lowest mean TSS concentration in the winter (2.2 ± 0.3 mg L^−1^; Table 3). Variation in storm size (measured as *Q*) corresponded to variation in mean TSS yield. As such, mean TSS yield was significantly greater in spring (36.8 ± 10.4 kg km^−2^ day^−1^) and fall (68.4 ± 19.7 kg km^−2^ day^−1^) compared to winter (1.2 ± 0.3 kg km^−2^ day^−1^) and summer (1.8 ± 1.0 kg km^−2^ day^−1^; ANOVA, *p* < 0.00; Table 3 and Table S4; Figure 2F). Bridge sites had the greatest mean TSS yield in the fall (117.8 ± 50.2 kg km^−2^ day^−1^) and culvert sites had the lowest mean TSS yield in the summer (0.1 ± 0.0 kg km^−2^ day^−1^; Table 3). Overall stormflow had greater TSS loss than baseflow (stormflow = 26.9 ± 6.8 kg km^−2^ day^−1^; baseflow = 0.4 ± 0.0 kg km^−2^ day^−1^; unpaired *t*‐test, *p* < 0.001; Figure S2).

### 3.2 Sediment yields were comparable US versus DS of road crossings

**FIGURE 3:**
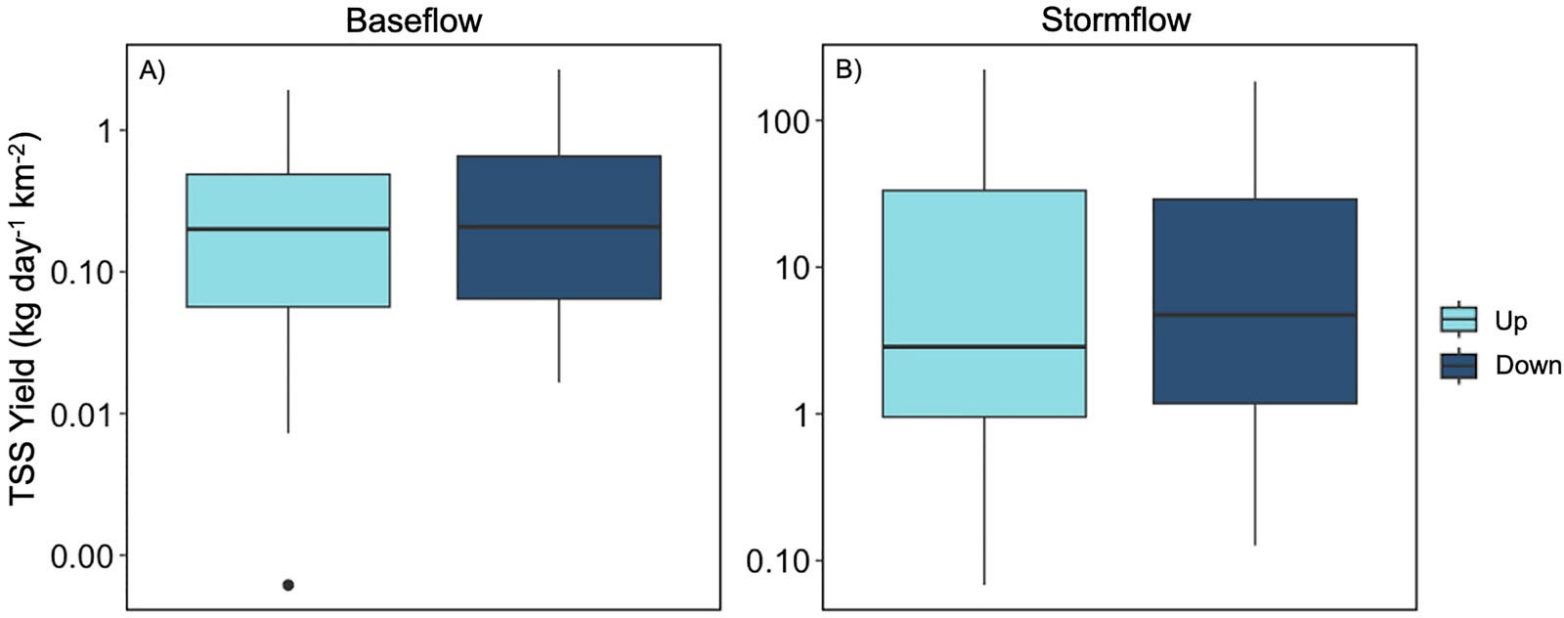
Upstream versus downstream average TSS yield during baseflow and stormflow. Note the *y*‐axis range varies based on flow condition and is shown on a log scale. For each site type box plot, the central thick horizontal line indicates the median of the distribution, the box limits represent the upper (Q3) and lower (Q1) quartiles, the whiskers extend to 1.5 times the interquartile range (IQR) from the box, and points beyond the whiskers denote outliers represented by black circles.

At baseflow, we did not document a difference in mean TSS yield US and DS of road crossings (US = 0.4 ± 0.1 kg km^−2^ day^−1^; DS = 0.4 ± 0.1 kg km^−2^ day^−1^; paired *t*‐test, *p* > 0.05; Figure 3A). This pattern was consistent at stormflow; we did not document any US versus DS differences (US = 26.7 ± 10.1 kg km^−2^ day^−1^; DS = 27.0 ± 9.3 kg km^−2^ day^−1^, paired *t*‐test, *p* > 0.05; Figure 3B). We did not document any seasonal variation in mean TSS yield US and DS of road crossings during baseflow or stormflow (paired test, *p* > 0.05 for both, Figure S3).

### 3.3 Road type crossing does not impact sediment yield

**FIGURE 4:**
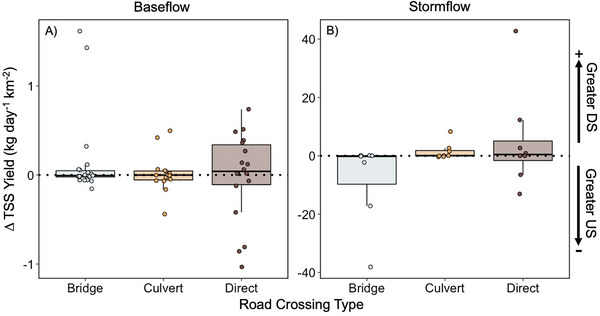
Average TSS yield difference at road crossing types during baseflow (A) and stormflow (B). TSS yield difference values above zero indicate greater downstream yield, and values below zero indicate greater upstream yield. For each road crossing type box plot, the central thick horizontal line indicates the median of the distribution, the box limits represent the upper (Q3) and lower (Q1) quartiles, and the whiskers extend to 1.5 times the interquartile range (IQR) from the box. Colored points on the box plot represent each sample with points beyond the whiskers denoting outliers.

In general, we did not find an effect of road crossing type on ΔTSS at baseflow (ANOVA, *p* > 0.05; Figure 4A) or stormflow (ANOVA, *p* > 0.05; Figure 4B). At baseflow, ΔTSS was near zero at bridge and culvert sites, indicating that TSS did not change as the stream went under the road (Figure 4A). However, direct crossings ΔTSS diverges from zero during baseflow, with greater DS TSS yield (Figure 4A). We observed different patterns in ΔTSS during stormflow compared to baseflow. We found ΔTSS was near zero at culvert sites during storms, while TSS yield was greater US of bridges and greater DS of direct stream crossings during storms (Figure 4B).

**FIGURE 5:**
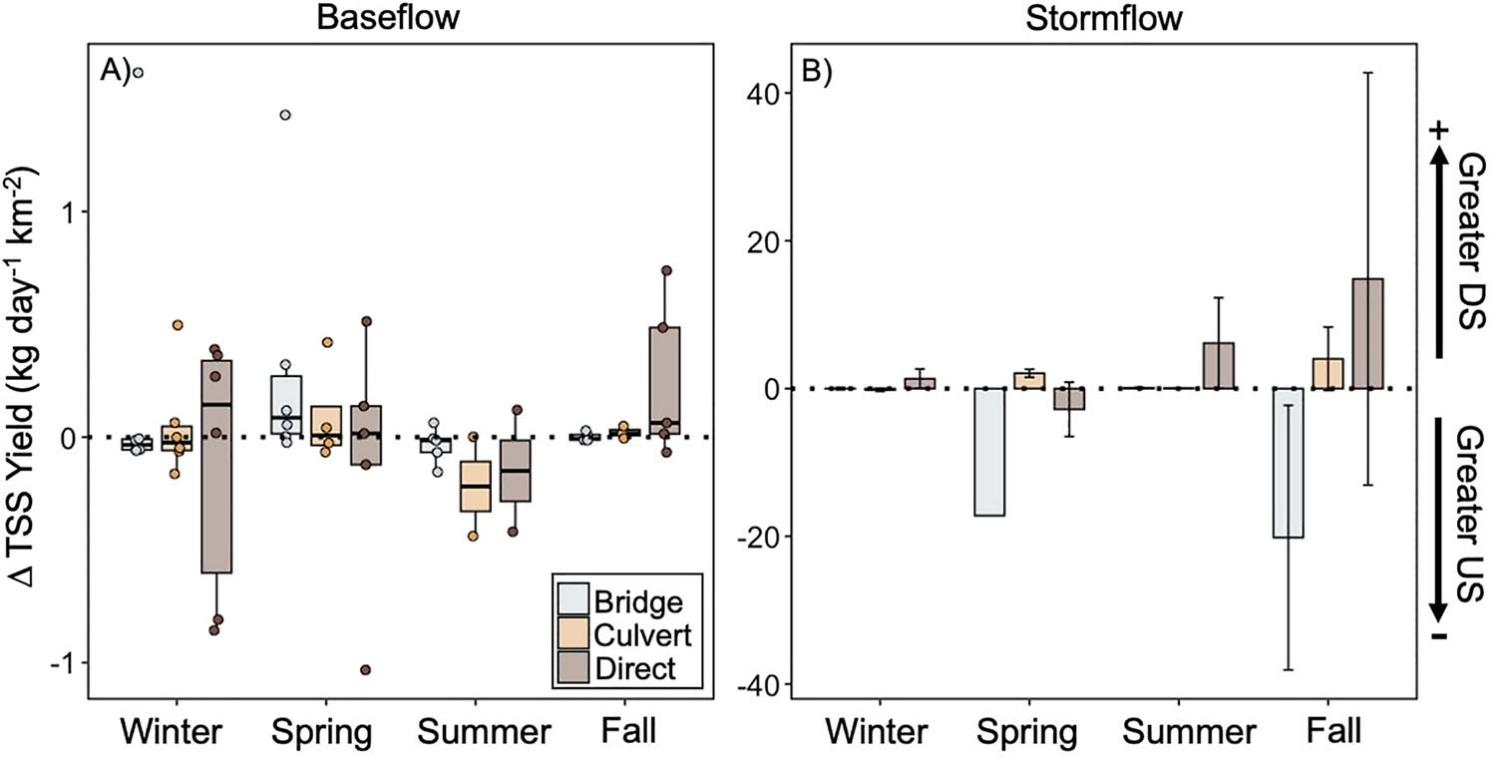
Average TSS yield difference at road crossing types across the seasons during baseflow and stormflow. TSS load difference values above zero indicate greater downstream yield, and values below zero indicate greater upstream yield. Bridge sites are shown in gray, culvert sites in orange, and direct crossings in brown. For each baseflow road crossing type box plot, the central thick horizontal line indicates the median of the distribution, the box limits represent the upper (Q3) and lower (Q1) quartiles, the whiskers extend to 1.5 times the interquartile range (IQR) from the box, and points beyond the whiskers denote outliers represented by colored points. Other colored points on the box plot represent each sample. For stormflow, values are depicted in a bar graph as some sites during seasons only have one observation (*n* = 1).

We did document seasonal variation in ΔTSS (Figure 5). During baseflow, winter and fall yields were similar to the overall mean (Figures 4 and 5A). At baseflow during the spring, TSS yields at bridge and culvert sites were greater DS (ΔTSS > 0), while ΔTSS was near zero at direct crossings (Figure 5A). During the summer, baseflow ΔTSS was higher US for all road crossing types (Figure 5A). In contrast, at stormflow, spring and fall were similar to the overall mean (Figures 4 and 5B). During the smaller winter and summer storms, we found that TSS yields were greater DS at direct road crossings (ΔTSS > 0), while ΔTSS were near zero for bridge and culvert sites (Figure 5B).

### 3.4 Land use showed ecologically meaningful trends with sediment load

**FIGURE 6:**
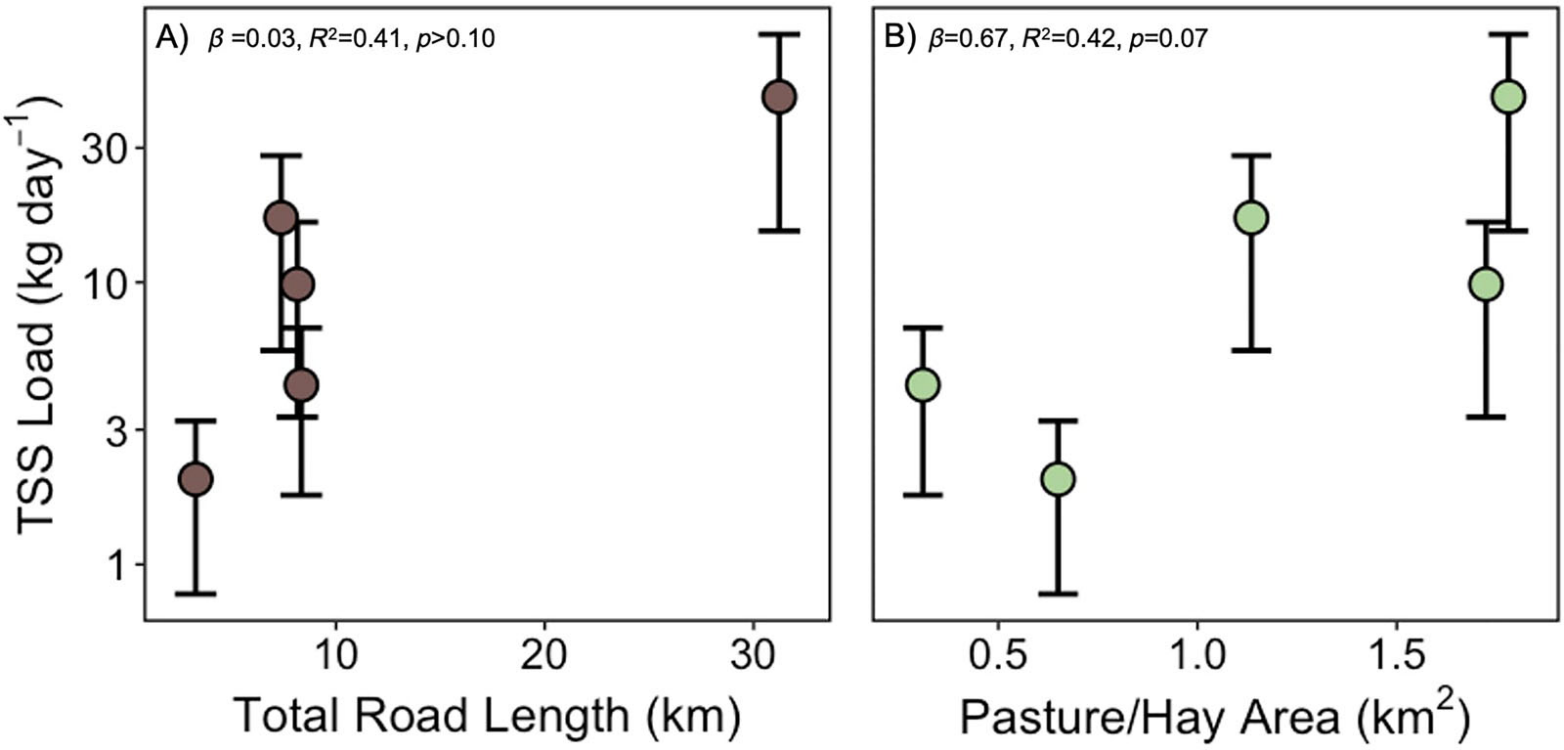
Average TSS load across land use variables for sites S2–S6: (A) total unpaved road length (km) and (B) area of pasture/hay (km^2^). Points show site means ± standard error (SE). Statistics from linear mixed effects models (*n* = 67 observations, five sites, flow condition as a covariate) appear in the figure panels. Note the *y*‐axis is shown on a log scale.

Total length of unpaved roads (LME, *β* = 0.03, *R* ^2 ^= 0.41, *p* > 0.1; Figure 6A) and pastureland area (LME, *β* = 0.67, *R* ^2 ^= 0.42, *p* = 0.07; Figure 6B) did not significantly impact mean TSS load for sites S2–S6 likely due to moderate collinearity between total length of unpaved roads and area of pastureland (*ρ* = 0.38, *p* = 0.002). Flow condition was found to be insignificant as an interaction term in the total unpaved road length interaction model (*p* > 0.1) and the pasture/hay area interaction model (*p* > 0.1), while absolute TSS loads differed between baseflow and stormflow (*p* < 0.001). Positive visual trends, large effect size, high *R* ^2^ for pastureland area, and moderate correlation between land use variables indicate ecologically meaningful effects of land use on TSS loss in Brush Creek.

## 4 DISCUSSION

### 4.1 Sediment dynamics are season and discharge dependent

The wet season is a key time of sediment delivery to streams (Schottler et al., 2014) as precipitation increases discharge and hydrologic connectivity with the landscape (Al‐Chokhacy et al., 2025; Danz et al., 2013; Rose & Karwan, 2021). For instance, we found that in Brush Creek, baseflow TSS yields were highest in the wet season (February–May), which correlated to the highest discharge during the sampling period (Figure 2A,C). Repeated precipitation events recharge groundwater, increasing baseflow in streams (Jung et al., 2021; Zomlot et al., 2015), which can then increase both mobilization of sediment from the streambed and erosion of streambanks (Baumgartner et al., 2022; Chen et al., 2001). In addition, increased soil saturation in the wet season can enhance overland flow delivering TSS to streams, which likely increases sediment delivery from unpaved roads. Although baseflow does not capture peak discharge periods associated with the greatest TSS loss, the seasonal patterns we documented highlight that seasonal changes in baseflow do indeed influence sediment transport (Duvert et al., 2011).

We found that stormflow events in Brush Creek resulted in higher mean TSS yields than baseflow (Figure S2), with higher discharge events in spring and fall contributing more to sediment loss (Figure 2F; Table 3; Danz et al., 2013). Stormflow is considered the dominant driver of sediment loss to streams, with individual storms acting as key contributors to overall transport (Bezak et al., 2016; Wolman & Miller, 1960). The combined effect of increased overland flow and higher velocities within the stream channel results in the erosion of streambeds and banks, widening channels, dislodging sediment, and transporting it further DS as sediment remains suspended (Fox et al., 2016; Schottler et al., 2014; Thorne & Osman, 1988; Tomer & Locke, 2011). Overland flow is a key mechanism in TSS runoff from the landscape with increased lateral connection as precipitation flows over source areas, such as unpaved roads, mobilizing sediments during storms to the stream (Jones et al., 2000; Krishnappan et al., 2009). Overall, our findings emphasize the importance of seasonal hydrologic variability in determining both the magnitude and timing of sediment transport in Brush Creek.

### 4.2 Road crossing type has a limited impact on sediment yield

We found that TSS yields US versus DS of a road crossing in Brush Creek did not differ, regardless of flow conditions (i.e., baseflow vs. stormflow), which contrasts our original hypothesis (Figures 3 and 4). Our findings also contrast with previous studies that found road crossings act as a source of fine sediment to streams, as sediment is entrained from roads, resulting in increased sediment loss DS (Arismendi et al., 2017; Lane & Sheridan, 2002; Thomaz et al., 2013). However, most of the previous studies were conducted in either forestry settings, at actively or newly constructed crossings, or under steep conditions (Arismendi et al., 2017; Lane & Sheridan, 2002; Thomaz et al., 2013). More aligned with our findings, Boggs et al. (2018) found no difference between US versus DS TSS yields in logging operation roads with varying road crossing types as BMPs at road crossings helped reduce additional sediment loss to streams.

It is important to note, however, that we documented the most variation in US and DS TSS yields at direct stream crossings (Figure 4). Sample et al. (1998) found that direct stream crossings or fords, particularly earthen fords without reinforcement or armored streambeds, generated high levels of TSS when vehicles disturbed the streambed sediments. To our knowledge, we did not collect any samples when vehicles disturbed the streambed. As such, sediment yields at direct crossings could have been significantly higher than the yields we captured following vehicle disturbance, emphasizing that highly trafficked direct stream crossings pose a significant risk for DS sediment loss. Overall, direct stream crossings may contribute to more TSS loss as a result of vehicle disturbance, highlighting the opportunity for the implementation of targeted BMPs at direct crossings, such as reinforced crossings and low water crossings (Keller & Sherar, 2003; Sample et al., 1998). Specifically, box and corrugated culverts have been found to maintain similar flow patterns to streams unimpacted by road crossings, while reducing the impact on the cross‐sectional area of the stream (Bouska et al., 2010).

Interestingly, we also documented greater US TSS yields at bridge sites during more intense spring and fall storms (Figure 5B). Scouring at high flows could have created deeper pools at bridges where sediment can be deposited (Kundu et al., 2024; Wellman et al., 2000). In fact, we found D_50_ was typically lower US of bridges (see Supporting Information for description; Table S5), indicating that US of bridges may indeed be depositional zones that can decrease fine sediment yields.

### 4.3 Increasing pastureland area suggests increases to sediment loads in rural watersheds

At the watershed scale, we found that areas of pasture/hay had an ecologically meaningful impact on sediment loads in Brush Creek (Figure 6). The positive trend observed, large effect size, and a high R^2^ in an ecological setting suggest that pastureland area in Brush Creek contributed to higher TSS load and warrants the need to investigate further with a larger sample size (Figure 6). Pasturelands can act as a source of TSS to streams via multiple mechanisms. Overgrazing can decrease vegetation and compact soil, leading to decreased soil infiltration and increased overland flow (Mulholland & Fullen, 1991; Pilon et al., 2017). Additionally, the application of poultry litter on pasturelands can be easily mobilized in runoff from storms, especially if applied close to a storm event (Katuwal et al., 2023; Schroeder et al., 2004). Direct livestock access to streams can also present an additional concern. When cows enter the stream, there is an increased risk of sediment loss via streambank and streambed sediment erosion (O'Callaghan et al., 2019). In Brush Creek, we observed cows at a direct road crossing site and documented TSS yields 13x higher than typical baseflow conditions (Figure S4). This emphasizes the importance of implementing BMPs on grazed pasturelands that reduce the direct access of livestock to streams (O'Callaghan et al., 2019).

Importantly, unpaved roads are one of the largest anthropogenic sources of fine sediments to small streams (Silliman & Toman, 2019). Sediment contributions from unpaved roads are amplified during precipitation events from overland flow (Ramos‐Scharrón & LeFevor, 2016), particularly when they are poorly maintained or not constructed using proper drainage BMPs (Silliman & Toman, 2019). In Brush Creek, 92.6% of roads are unpaved, highlighting a potential risk if roads are not properly maintained. Additionally, we found moderate correlation between unpaved road length and pastureland area emphasizing the need for BMPs to consider both features. Overall, it is important to track key periods of TSS transport, such as storms, to implement targeted BMPs, as well as to inform water quality managers on periods of water quality risk.

## 5 CONCLUSIONS

We have documented several key controls on TSS yields and loads in Brush Creek in the current study: seasonality, stormflow, the length of unpaved roads, and the area of pastureland. Contrasting our original hypothesis, road crossings type had less impact on sediment yields than anticipated, suggesting that implementing road crossing BMPs, such as culverts and bridges, may be effective in reducing local‐scale TSS loss. Importantly, we measured up to 13 tonnes day^−1^ of sediment loss on the falling limb of the largest storm event. This suggests that overall sediment loss during high‐intensity events could be even greater when accounting for peak flow. Our findings underscore the importance of implementing targeted BMPs to manage sediment loss during storms.

While paving roads may seem like a simple solution, increased impervious surface cover increases runoff of high discharge flows, exacerbating erosion from road margins, ditches, and stream banks (Pappas et al., 2008; Yu et al., 2021). When properly maintained, unpaved roads can actually increase water infiltration, decreasing high‐velocity runoff during storms (Hawks et al., 2022; Keller & Sherar, 2003; Silva et al., 2021). The Beaver Lake Watershed Protection Strategy in Northwest Arkansas estimated that implementing unpaved road BMPs could decrease sediment loads by 810 tons year^−1^ to Beaver Lake Reservoir, a key drinking water source (Beaver Watershed Alliance, 2012). In addition, properly timed BMPs on pasturelands, such as pasture aeration and poultry litter incorporation, can decrease sediment loss by reducing compacted soil and increasing infiltration (Ashworth et al., 2021; Lambert et al., 2020). Given the extensive network of unpaved roads and large area of pasturelands in Arkansas and rural areas generally, maintaining well‐functioning BMPs for unpaved roads and pasturelands is critical. These BMPs must withstand increased traffic volumes in rural areas and unpredictable precipitation patterns driven by climate change, which can both enhance erosion and lead to BMP failure. As such, proactive management is essential to protect water quality and ensure the long‐term sustainability of critical water resources.

## AUTHOR CONTRIBUTIONS

**Kathleen J. Cutting**: Conceptualization; data curation; formal analysis; investigation; methodology; visualization; writing—original draft. **Shannon L. Speir**: Conceptualization; funding acquisition; project administration; resources; supervision; writing—review and editing. **Alana G. Strauss**: Conceptualization; data curation; methodology. **Karessa G. De La Paz**: Data curation. **Caroline G. T. Anscombe**: Data curation; writing—review and editing.

## CONFLICT OF INTEREST STATEMENT

The authors declare no conflicts of interest.

## Supporting information



The supplemental materials include soil type data, statistical test results, and mean particle size methods and results, along with additional figures showing the ecoregions of the study site, sediment yield across flow condition, seasons, and sediment yield from cow stream disturbance.

## Data Availability

The sediment and discharge data utilized for this study are available at HydroShare at https://doi.org/10.4211/hs.ce4f9ad6285d47fbb67e100c7225c7af. These data are publicly available for download.
