# Supplementary material for: Watershed‐scale controls outweigh local crossing effects on sediment loss from unpaved roads
Source: J Environ Qual. 2026 Jan 21;55(1):e70138. doi: 10.1002/jeq2.70138 (PMC12823549; doi:10.1002/jeq2.70138)
Supplement: Supplementary file 1 — The supplemental materials include soil type data, statistical test results, and mean particle size methods and results, along with additional figures showing the ecoregions of the study site, sediment yield across flow condition, seasons, and sediment yield from cow stream disturbance. [file JEQ2-55-0-s001.docx]

**Supplemental Information**

**Watershed-scale controls outweigh local crossing effects on sediment loss from unpaved roads**

Kathleen J. Cutting*, Shannon L. Speir^1^, Alana G. Strauss^2^, Karessa G. De la Paz^1^, Caroline G. T. Anscombe^1^

Table S1. Soil type percentages throughout the Brush Creek watershed listed from greatest to least.

| Soil Type | Percent |
| --- | --- |
| \| Enders-Leesburg complex, 8 to 20 percent slopes \| \| --- \| \| Enders-Leesburg complex, 20 to 40 percent slopes \| \| Noark very gravelly silt loam, 20 to 40 percent slopes \| \| Nixa very gravelly silt loam, 3 to 8 percent slopes \| \| Clarksville very gravelly silt loam, 20 to 50 percent slopes \| \| Tonti gravelly silt loam, 3 to 8 percent slopes \| \| Enders stony loam, 3 to 15 percent slopes \| \| Nixa very gravelly silt loam, 8 to 12 percent slopes \| \| Linker loam, 3 to 8 percent slopes \| \| Clarksville extremely gravelly silt loam, 12 to 60 percent slopes \| \| Leadvale loam, 3 to 8 percent slopes \| \| Mountainburg stony loam, 3 to 20 percent slopes \| \| Steprock gravelly loam, 3 to 8 percent slopes \| \| Secesh gravelly silt loam, 0 to 3 percent slopes, occasionally flooded \| \| Noark very gravelly silt loam, 12 to 20 percent slopes \| \| Nixa very gravelly silt loam, 3 to 8 percent slopes \| \| Enders-Leesburg complex, 20 to 40 percent slopes \| \| Cleora fine sandy loam, occasionally flooded \| \| Enders-Leesburg complex, 8 to 20 percent slopes \| \| Baxter very gravelly silt loam, 20 to 45 percent slopes \| \| Ceda cobbly fine sandy loam, 0 to 3 percent slopes, frequently flooded \| \| Mountainburg very stony loam, 20 to 50 percent slopes \| \| Captina silt loam, 1 to 3 percent slopes \| \| Captina silt loam, 3 to 6 percent slopes, eroded \| \| Nella-Steprock-Mountainburg very stony loams, 20 to 40 percent slopes \| \| Steprock stony loam, 3 to 12 percent slopes \| \| Elsah very cherty silt loam, occasionally flooded \| \| Leesburg gravelly loam, 8 to 12 percent slopes \| \| Peridge silt loam, 3 to 8 percent slopes \| \| Apison loam, 3 to 8 percent slopes, eroded \| \| Nixa very gravelly silt loam, 8 to 12 percent slopes \| \| Allegheny stony loam, 12 to 40 percent slopes (leesburg) \| \| Enders gravelly loam, 3 to 8 percent slopes \| \| Razort silt loam, occasionally flooded \| \| Leesburg stony loam, 8 to 12 percent slopes \| \| Enders gravelly loam, 8 to 15 percent slopes \| \| Guin very gravelly silt loam, 3 to 8 percent slopes (clarksville) \| \| Peridge silt loam, 1 to 3 percent slopes \| \| Fayetteville fine sandy loam, 3 to 8 percent slopes eroded \| \| Allen loam, 3 to 8 percent slopes \| \| Johnsburg silt loam, 0 to 2 percent slopes \| \| Hector-Mountainburg stony fine sandy loams, 3 to 40 percent slopes, rocky \| \| Sloan silt loam \| \| Allegheny gravelly loam, 3 to 8 percent slopes, eroded (leesburg) \| \| Moko very stony silt loam, very rocky, 12 to 40 percent slopes \| \| Elsah gravelly soils (ceda) \| \| Captina silt loam, 1 to 3 percent slopes \| \| Britwater gravelly silt loam, 3 to 8 percent slopes \| \| Nella stony loam, 8 to 20 percent slopes \| \| Nella gravelly loam, 8 to 12 percent slopes \| \| Noark very gravelly silt loam, 8 to 12 percent slopes \| \| Mountainburg gravelly loam, 3 to 12 percent slopes \| \| Allegheny gravelly loam, 8 to 12 percent slopes, eroded (leesburg) \| \| Fayetteville fine sandy loam, 8 to 12 percent slopes, eroded \| \| Enders stony loam, 3 to 15 percent slopes \| \| Healing silt loam, 1 to 3 percent slopes, rarely flooded \| \| Cleora fine sandy loam \| \| Allegheny stony loam, 8 to 12 percent slopes (leesburg) \| \| Nella gravelly loam, 12 to 20 percent slopes \| \| Mayes silty clay loam, 0 to 1 percent slopes \| \| Johnsburg silt loam, 0 to 2 percent slopes \| \| Baxter very gravelly silt loam, 12 to 20 percent slopes \| \| Linker loam, 3 to 8 percent slopes, eroded \| \| Waben very gravelly silt loam, 3 to 12 percent slopes \| \| Leesburg gravelly loam, 3 to 8 percent slopes \| \| Nella gravelly loam, 3 to 8 percent slopes \| \| Apison gravelly loam, 3 to 8 percent slopes, eroded \| \| Linker loam, 1 to 3 percent slopes \| \| Allegheny gravelly loam, 3 to 8 percent slopes (leesburg) \| | \| 20.09 \| \| --- \| \| 7.19 \| \| 7.18 \| \| 6.25 \| \| 5.99 \| \| 4.32 \| \| 4.31 \| \| 4.22 \| \| 3.30 \| \| 3.21 \| \| 2.84 \| \| 2.41 \| \| 2.15 \| \| 2.14 \| \| 1.84 \| \| 1.82 \| \| 1.36 \| \| 1.33 \| \| 1.22 \| \| 1.08 \| \| 1.00 \| \| 0.88 \| \| 0.75 \| \| 0.70 \| \| 0.67 \| \| 0.64 \| \| 0.61 \| \| 0.54 \| \| 0.53 \| \| 0.48 \| \| 0.48 \| \| 0.46 \| \| 0.45 \| \| 0.45 \| \| 0.43 \| \| 0.42 \| \| 0.42 \| \| 0.40 \| \| 0.39 \| \| 0.37 \| \| 0.37 \| \| 0.36 \| \| 0.33 \| \| 0.33 \| \| 0.30 \| \| 0.28 \| \| 0.28 \| \| 0.25 \| \| 0.25 \| \| 0.22 \| \| 0.20 \| \| 0.19 \| \| 0.15 \| \| 0.15 \| \| 0.12 \| \| 0.12 \| \| 0.11 \| \| 0.09 \| \| 0.09 \| \| 0.08 \| \| 0.08 \| \| 0.06 \| \| 0.06 \| \| 0.06 \| \| 0.04 \| \| 0.03 \| \| 0.03 \| \| 0.02 \| \| 0.02 \| |

Table S2. P-values from Tukey test statistics for seasonal Q based on significant one-way ANOVA tests for baseflow (p<0.01), and stormflow (p<0.001); logged Q and season. Significant p-values are bolded.

| **Baseflow** | | | | | | |  | | **Stormflow** | | | | | | | | | | |
| --- | --- | --- | --- | --- | --- | --- | --- | --- | --- | --- | --- | --- | --- | --- | --- | --- | --- | --- | --- |
|  | Winter | Spring | Summer | | Fall |  | |  | | Winter | | Spring | | Summer | | Fall | |  |  |
| Winter | NA | 0.98 | | **0.00** | 0.58 | |  | | Winter | | NA | | **0.01** | | 0.31 | | **0.00** | |  |
| Spring | 0.98 | NA | | **0.02** | 0.83 | |  | | Spring | | **0.01** | | NA | | **0.00** | | 0.93 | |  |
| Summer | **0.00** | **0.02** | | NA | 0.14 | |  | | Summer | | 0.31 | | **0.00** | | NA | | **0.00** | |  |
| Fall | 0.58 | 0.83 | | 0.14 | NA | |  | | Fall | | **0.00** | | 0.93 | | **0.00** | | NA | |  |
|  |  |  | |  |  | |  | |  | |  | |  | |  | |  | |  |

**Table S3.** P-values from Tukey test statistics for for seasonal TSS concentrations based on significant one-way ANOVA tests for baseflow (p<0.001), and stormflow (p<0.01); logged TSS concentrations and season. Significant p-values are bolded.

| **Baseflow** | | | | | | |  | | **Stormflow** | | | | | | | | | | |
| --- | --- | --- | --- | --- | --- | --- | --- | --- | --- | --- | --- | --- | --- | --- | --- | --- | --- | --- | --- |
|  | Winter | Spring | Summer | | Fall |  | |  | | Winter | | Spring | | Summer | | Fall | |  |  |
| Winter | NA | **0.00** | | **0.00** | 0.16 | |  | | Winter | | NA | | 0.11 | | **0.03** | | **0.03** | |  |
| Spring | **0.00** | NA | | 0.99 | **0.00** | |  | | Spring | | 0.11 | | NA | | 0.97 | | 0.97 | |  |
| Summer | **0.00** | 0.99 | | NA | **0.00** | |  | | Summer | | **0.03** | | 0.97 | | NA | | 1.00 | |  |
| Fall | 0.16 | **0.00** | | **0.00** | NA | |  | | Fall | | **0.03** | | 0.97 | | 1.00 | | NA | |  |

**Table S4.** P-values from Tukey test statistics for seasonal TSS yields based on significant one-way ANOVA tests for baseflow (p<0.001), and stormflow (p<0.001); logged TSS yields and season. Significant p-values are bolded.

| **Baseflow** | | | | | | |  | | **Stormflow** | | | | | | | | | | |
| --- | --- | --- | --- | --- | --- | --- | --- | --- | --- | --- | --- | --- | --- | --- | --- | --- | --- | --- | --- |
|  | Winter | Spring | Summer | | Fall |  | |  | | Winter | | Spring | | Summer | | Fall | |  |  |
| Winter | NA | 0.41 | | **0.06** | **0.04** | |  | | Winter | | NA | | **0.00** | | 0.91 | | **0.00** | |  |
| Spring | 0.41 | NA | | **0.00** | **0.00** | |  | | Spring | | **0.00** | | NA | | **0.00** | | 0.55 | |  |
| Summer | **0.06** | **0.00** | | NA | 0.10 | |  | | Summer | | 0.91 | | **0.00** | | NA | | **0.00** | |  |
| Fall | **0.04** | **0.00** | | 0.10 | NA | |  | | Fall | | **0.00** | | 0.55 | | **0.00** | | NA | |  |

**Methods**

*Median Particle Size*

We measured median particle size (D_50_) once over the study duration both upstream and downstream of road crossing at each sampling site. In areas without bedrock within the transect, we used a gravelometer at three transects at each sampling location. At each transect, we took approximately 10 measurements across the stream at three locations: the sampling site, 5 m upstream of the sampling site, and 5 m downstream of the sampling site (Tavernini & Richardson, 2020). When bedrock was present within the transect, we measured the bedrock length to estimate the percent of bedrock in the transect. Median particle size and standard error of the median were averaged at each site to find overall D_50_.

**Results**

*Mean Particle Size*

We used mean particle size measurements (D_50_) to further explore site sediment characteristics. We found that bridge sites generally had smaller particle size upstream of the road crossing.

Table S5. Median particle size (D_50_) and the standard error of the median [SE(M)]; the percent of bedrock at each sampling site.

| **Site** | **D_50_** | **Bedrock** |
| --- | --- | --- |
|  | mm | % |
| S1 down | 27.3 ± 9.1 | 0 |
| S1 up | 24.6 ± 5.3 | 0 |
| S2 down | 49.2 ± 50 | 0 |
| S2 up | 28.7 ± 21.4 | 0 |
| S3 down | 45.5 ± 89.4 | 63 |
| S3 up | 14 ± 7.4 | 63 |
| S4 down | 27 ± 16.8 | 0 |
| S4 up | 49.8 ± 31.1 | 0 |
| S5 down | 18.9 ± 12.1 | 38 |
| S5 up | 28.8 ± 26.6 | 0 |
| S6 down | 6.7 ± 4.6 | 0 |
| S6 up | 11.7 ± 12 | 0 |

**References**

1. Tavernini, D. A., and J. S. Richardson. 2020. Effects of tributary size on the resource supply and physical habitat at tributary junctions along two mainstem rivers. Canadian Journal of Fisheries and Aquatic Sciences 77:1393–1408.


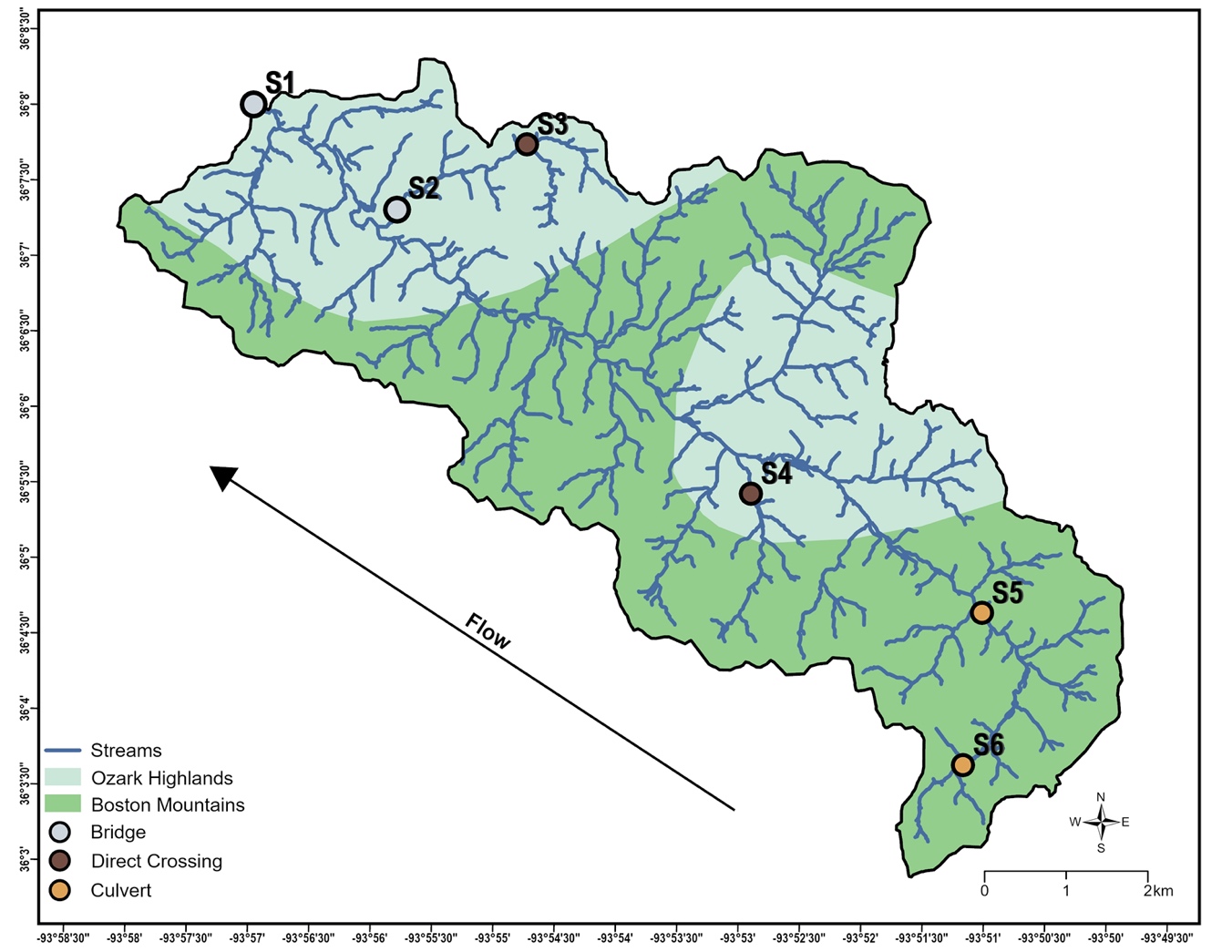


Figure S1. The Brush Creek watershed, with site S1 as the outlet and increasing in number moving upstream. Bridge crossings are shown in grey, direct crossings in brown, and culvert crossings in orange. The ecoregions of the Brush Creek are shown by the lighter green (Ozark Highlands) and darker green (Boston Mountains).

**
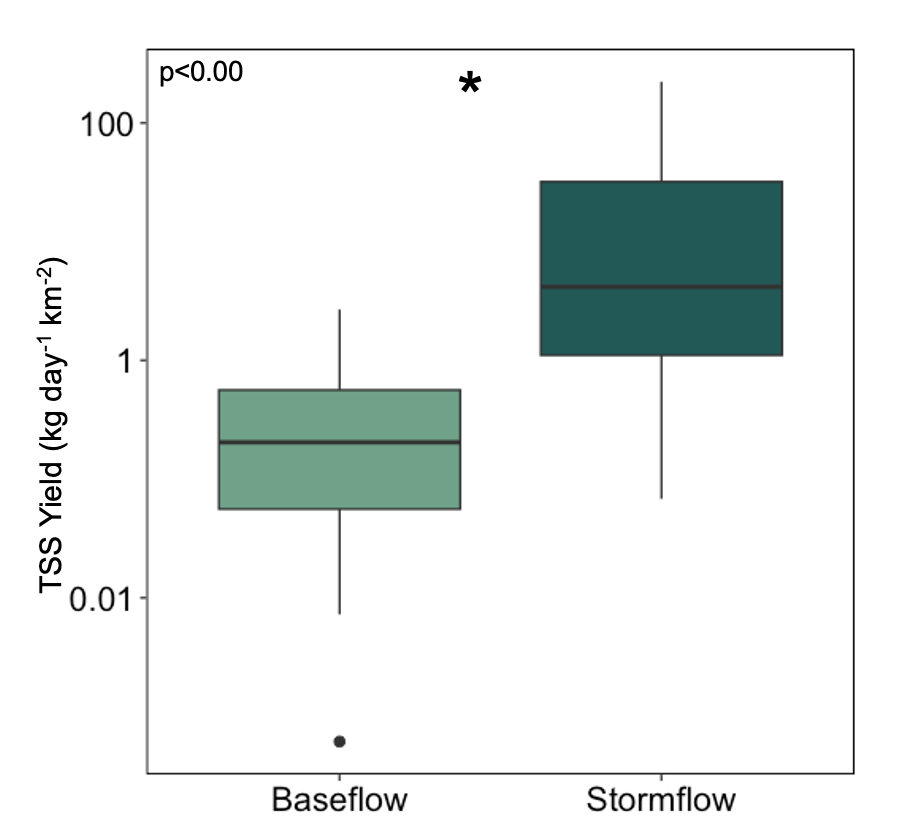
**

Figure S2. Average TSS yield during baseflow (light green) and storm flow (dark green). Y-axis is log transformed for normality. For each flow condition box plot, the central thick horizontal line indicates the median of the distribution, the box limits represent the upper (Q3) and lower (Q1) quartiles, the whiskers extend to 1.5 times the interquartile range (IQR).

**
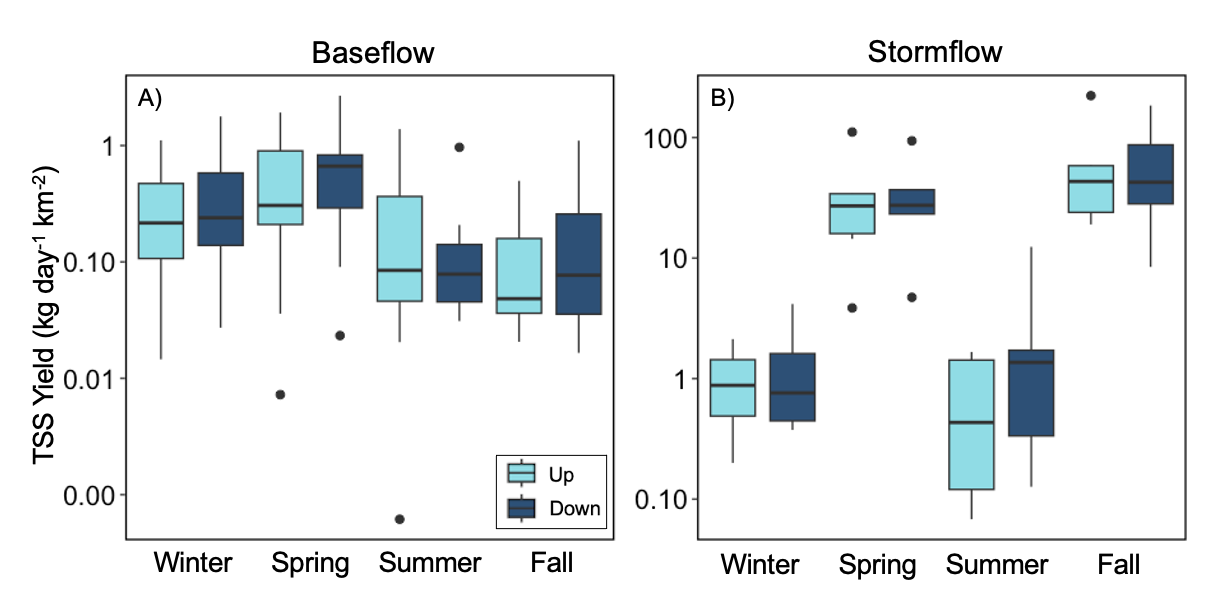
**

Figure S3. Mean TSS yield during baseflow (A) and stormflow (B), between upstream (light blue) and downstream (dark blue) sampling locations. Y-axes are log transformed. For each season box plot, the central thick horizontal line indicates the median of the distribution, the box limits represent the upper (Q3) and lower (Q1) quartiles, the whiskers extend to 1.5 times the interquartile range (IQR) from the box, and points beyond the whiskers denote outliers represented by black circles.

**
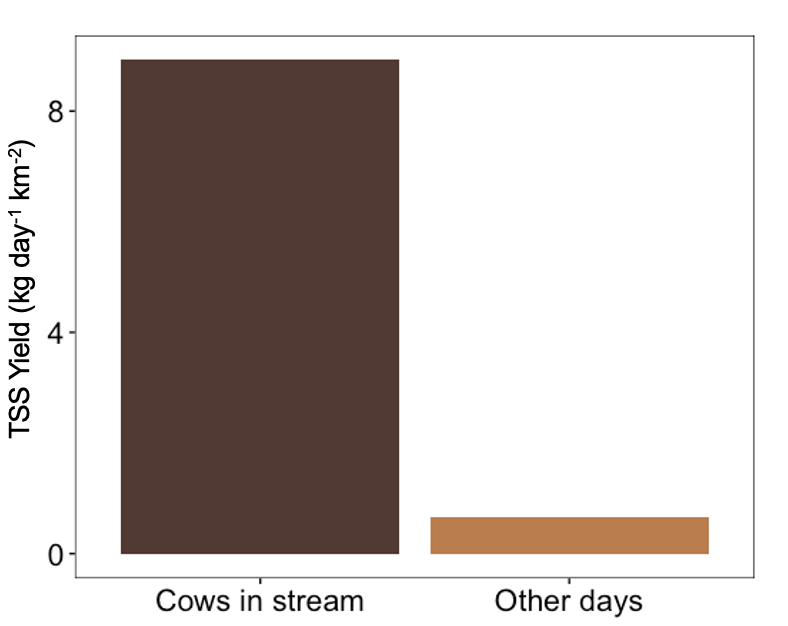
**

Figure S4. Sediment yield at a downstream direct stream crossing site when cows were in the stream versus when they were not at the time of baseflow sampling. The “cow” TSS yield is representative of one day, where the other days are the average of the TSS yield throughout the sampling period.
